# Cell cycle regulated transcription: from yeast to cancer

**DOI:** 10.12688/f1000research.8111.1

**Published:** 2016-05-12

**Authors:** Christopher J. McInerny

**Affiliations:** 1 School of Life Sciences, University of Glasgow, Scotland, UK

**Keywords:** Cell cycle, yeast, transcription, Cell proliferation, cancer

## Abstract

Recent studies have revealed exciting new functions for forkhead transcription factors in cell proliferation and development. Cell proliferation is a fundamental process controlled by multiple overlapping mechanisms, and the control of gene expression plays a major role in the orderly and timely division of cells. This occurs through transcription factors regulating the expression of groups of genes at particular phases of the cell division cycle. In this way, the encoded gene products are present when they are required. This review outlines recent advances in our understanding of this process in yeast model systems and describes how this knowledge has informed analysis in more developmentally complex eukaryotes, particularly where it is relevant to human disease.

## Introduction

The accurate duplication of a cell is essential for successful cell proliferation. Many cellular mechanisms control the cell division cycle, and some operate in parallel and show redundancy. This “belt and braces” approach contributes to the incredible fidelity of the process. The control mechanisms range from changes in protein activity (for example, through modifications in phosphorylation status) to changes in protein stability or localisation.

Another major level of control is through the regulation of gene transcription, where genes or groups of genes are transcribed at particular cell cycle times in order to ensure that protein products are produced when they are required. In some cases, the protein products have structural or enzymatic functions specific to a certain cell cycle phase. In other cases, they have regulatory roles such as activating other proteins important for cell division or controlling the transcription of genes required later in the cell cycle. There are also a few examples of protein products whose presence at the “wrong” cell cycle time is deleterious to the cell.

## Yeasts

**Figure 1.**
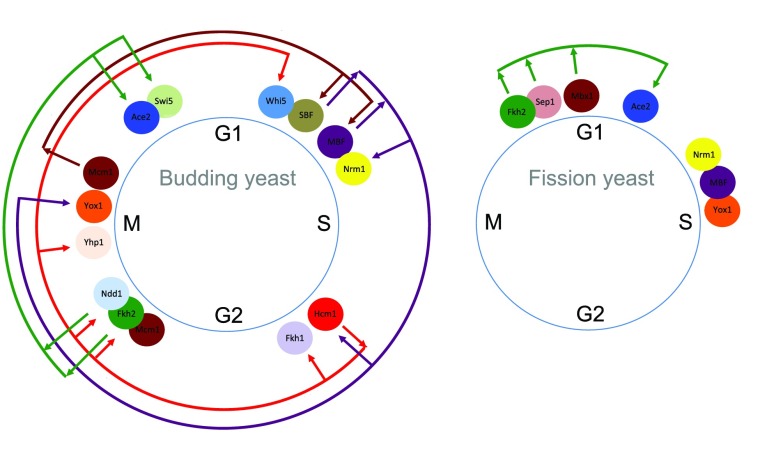
Networks of transcription factors controlling gene expression during the cell cycles of budding and fission yeasts. In each yeast species, the principal transcription factors are shown, and the downstream transcription factor(s) that they regulate, either positively or negatively, are indicated by arrows. Where similar transcription factors are present in the two species, they are shown in the same colour. In budding yeast, a reasonably complete network exists, whereby consecutive regulation of transcription factors encompasses the whole cell cycle. In contrast, the network in fission yeast is more limited.

Regulation of gene transcription through the cell division cycle has been studied most extensively in two yeast species: the budding yeast *Saccharomyces cerevisiae* and the fission yeast *Schizosaccharomyces pombe* ^1– 3^ ( Figure 1). In both yeasts, groups of genes are transcribed at different cell cycle phases. The expression of each group of genes is controlled by a different transcription factor. Each transcription factor binds to DNA enhancer sequences that are present only in the promoter regions of genes transcribed at that cell cycle phase, ensuring cell cycle-specific gene transcription.

In budding yeast, where the most comprehensive transcription network has been elucidated and described, there exists a series of transcription factors that stimulate or repress the expression of consecutive groups of genes ^1^ ( Figure 1). In some cases, the genes encode transcription factors that stimulate or repress the expression of the next group of gene transcription in the cell cycle. Thus, the groups of gene expression can be functionally linked, and this linkage encompasses the whole cell cycle. In fission yeast, a more limited network has been described, and three groups of gene expression are observed ( Figure 1); in only one case is there evidence that one group of gene expression controls the expression of the next group ^2, 3^.

Strikingly, however, a number of these transcription factors are evolutionarily conserved with related proteins found in the two yeast species. Furthermore, these same transcription factors have been identified in more developmentally complex eukaryotes (including humans) where they too control cell cycle gene expression ^1– 3^. Examples include (1) the forkhead transcription factors which control expression of two groups of genes in the cell cycle, one during the mitotic (M) phase and the other at the end of the M phase and the beginning of the Gap 1 (G _1_) period, and (2) the MBF-Nrm1 transcription factor complex which regulates gene expression at the end of the G _1_ period and the start of the synthesis (S) phase. The remainder of this review will focus on these two systems, first by describing them in more detail in each model yeast and then by explaining their function in human cells and in disease.

## Forkhead transcription factor gene regulation

The role of forkhead transcription factors in cell cycle-regulated gene expression was first described in budding yeast. The forkhead transcription factor Fkh2, in combination with the MADS box protein Mcm1, controls gene expression during M phase ^1– 5^ ( Figure 1). In addition to regulating the expression of M phase genes, Fkh2-Mcm1 controls the activity of the transcription factors Ace2 and Swi5, which regulate gene expression later in the cell cycle during G _1_.

Similar mechanisms have been identified in fission yeast, where the forkhead transcription factor Fkh2, in combination with the MADS box protein Mbx1, regulates M-G _1_ gene expression as well as the subsequent group of gene expression in G _1_ through the regulation of the Ace2 transcription factor ^6, 7^ ( Figure 1).

Such conservation of control mechanisms between two distantly related yeasts suggests that forkhead transcription factors are important. Consistent with this suggestion, similar control processes have been identified in more developmentally complex eukaryotes. For example, the human forkhead transcription factors FOXO1 and FOXO2 regulate cell cycle gene transcription that is critical for proliferation ^8, 9^. Furthermore, the medical importance of FOXO1 and FOXO2 has been established, as these transcription factors have roles in a wide range of human medical conditions, including cancer, obesity, diabetes, autoimmune disease, and ageing ^10– 14^. Recently, the formation of blood vessels through the control of the metabolism and proliferation of vascular endothelial cells has been shown to be under the control of FOXO1 ^15^. Indeed, forkhead transcription factors are now seen as promising therapeutic targets for a wide variety of conditions ^16, 17^.

## MBF-Nrm1 gene regulation

As one of the first cell cycle transcription factors to be identified in yeasts, the MBF complex has a long history ^1– 3^. In budding yeast, it is composed of Swi4 and Mbp1 and controls the expression of a large group of genes in late G _1_ and early S phase ( Figure 1). MBF in fission yeast is composed of related proteins (including Cdc10, Res1, and Res2) and similarly controls gene expression at G _1_/S. MBF in both yeasts is controlled by Nrm1 ^18– 21^.

The human functional equivalent to MBF is E2F, which, along with pocket proteins such as the retinoblastoma protein Rb, controls the expression of genes at G _1_/S ^9, 22^. The expression of these genes is deregulated in many types of cancer, as they allow cancer cells to divide in the absence of growth factors, thereby making them insensitive to signals that normally inhibit growth ^23, 24^. E2F family members are now potential therapeutic targets for certain malignancies ^25^.

Recent experiments have revealed further mechanisms by which deregulated gene transcription can contribute to abnormal cell cycles, the hallmark of cancer and tumorigenesis. Caetano *et al*. ^26^ have shown that in fission yeast deregulation of G _1_/S gene expression increases DNA replication errors. This demonstrates that faulty transcription of certain genes under the control of MBF can have a profound impact on genome stability. It seems likely that similar mechanisms operate in human cells and that these may be possible targets for therapeutics.

## Future challenges

The examples described here show how yeast model systems have been used to identify proteins that control the cell division cycle in all eukaryotes, including humans. That these proteins have subsequently been shown to be useful therapeutic targets suggests that this avenue of study will continue to be useful in the future. It seems likely that more transcription factors with cell cycle-specific functions remain to be identified and, moreover, that yeasts will continue to offer an excellent system in which to discover and characterise them.
